# Three-Dimensional Hierarchical Porous Carbons Derived from Betelnut Shells for Supercapacitor Electrodes

**DOI:** 10.3390/ma14247793

**Published:** 2021-12-16

**Authors:** Arjunan Ariharan, Sung-Kon Kim

**Affiliations:** School of Chemical Engineering, Jeonbuk National University, Jeonju 54896, Korea; ariharanmsc87@gmail.com

**Keywords:** carbon material, biomass-derived carbon, porous structure, supercapacitors, energy and power density

## Abstract

Electrochemical energy storage (EES) systems are attracting research attention as an alternative to fossil fuels. Advances in the design and composition of energy storage materials are particularly significant. Biomass waste-derived porous carbons are particularly suitable for use in EES systems as they are capable of tuning pore networks from hierarchical porous structures with high specific surface areas. These materials are also more sustainable and environmentally friendly and less toxic and corrosive than other energy storage materials. In this study, we report the creation of a three-dimensional hierarchical porous carbon material derived from betelnut shells. The synthesized three-dimensional (3D) hierarchical porous carbon electrode showed a specific capacitance of 290 F g^−1^ using 1 M KOH as an electrolyte at a current density of 1 A g^−1^ in three-electrode systems. Moreover, it offered a high charge/discharge stability of 94% over 5000 charge–discharge cycles at a current density of 5 A g^−1^. Two-electrode symmetric systems show a specific capacitance of 148 F g^−1^, good cyclic stability of 90. 8% for 5000 charge-discharge cycles, and high energy density of 41 Wh Kg^−1^ at the power density of 483 W Kg^−1^ in aqueous electrolyte.

## 1. Introduction

The depletion of fossil fuels is one of the critical global energy and environmental problems. To tackle this issue, the research community must explore new, sustainable, and environment-friendly energy resources that can be substituted for fossil fuels [1]. Indeed, it appears that we are going in the direction of an electricity-based energy economy [2]. Supercapacitors (SCs) are one of promising EESs, because they show promise for use in portable devices and electric vehicles due to their outstanding properties, including a large power density, a long cycle life, a quick charge-discharge rate, and relatively low cost [3,4]. SCs are expected to have an identical importance to batteries for forthcoming energy storage technologies. However, the comparatively low energy density of SCs limits their practical application on a large scale. Green, sustainable, cost-effective, and eco-friendly electrode materials with fast ion/electron transport and a high surface area are now required by next-generation EES systems [5,6,7]. Hence, it is essential to search for innovative electrode materials to enhance the energy density of SCs. Carbon materials can play the role of electrode for SCs, as they have a high specific surface area, are cost-effective, and possess good electrical conductivity, abundant active sites, and intrinsic physicochemical stability [8,9,10]. Carbon materials demonstrate electrical double layer capacitor (EDLC)-type behavior that relies on the physical electrostatic attraction between ions and electrode surfaces [11,12]. Examples include graphene, carbon nanotubes, carbon onions, carbon spheres, carbon nanofibers, carbon aerogels, carbide-derived carbons, and activated carbon [13,14,15,16,17,18,19,20,21]. Unfortunately, these materials require a complicated synthesis process under relatively hard experimental conditions, and the prepared materials often show unordered morphological structures that impair industrial-level mass production [22,23]. The primary reason to produce electrode materials with different dimensional porous nanostructures is to increase the specific surface area and ion transport, to as this improves the electrochemical performances of SCs [11].

Carbon materials derived from natural or biomass/biomass wastes have potential as electrodes due to their renewable nature, extensive availability, and environmental friendliness [24,25]. Biomass-derived carbon materials naturally have a various shape of zero- to three-dimensional (3D) porous structures that can be formed on their own; these properties vary from different types of biomass precursors [6]. Some of previous SC studies reported the potential of using biomass as an electrode, but the majority of them used the activation process by using different types of activation agent such as KOH. The examples of such biomasses include bamboo [26], willow catkin [27], and eggshell membranes [28]. These types of chemical activation agents may be harmful; therefore, it is crucial to take more mild synthetic routes without using hazardous activation agents to produce high-performance porous carbon electrodes for SCs derived from renewable biomass precursors.

The betelnut tree is one of the significant plants in Southeast Asia; hundreds of millions of betelnuts are consumed every year worldwide. This usage of betelnut leads to the increase of betelnut-shell waste. Motivated by “waste to wealth”, we discuss 3D hierarchical porous carbon derived from renewable and sustainable betelnut shell for the utilization as a potential electrode material for high performance SCs. We also demonstrate a simple route to synthesize carbon material without the usage of a hazardous chemical activation process. Interestingly, the synthesized 3D hierarchical porous carbon showed a significant specific capacitance and excellent cyclic stability. This biomass-derived electrode allows for the expansion of next-generation SCs with enhanced specific capacitance and energy density.

## 2. Materials and Methods

### 2.1. Preparation of 3D Hierarchical Porous Carbon from Biomass Waste

**Figure 1:**
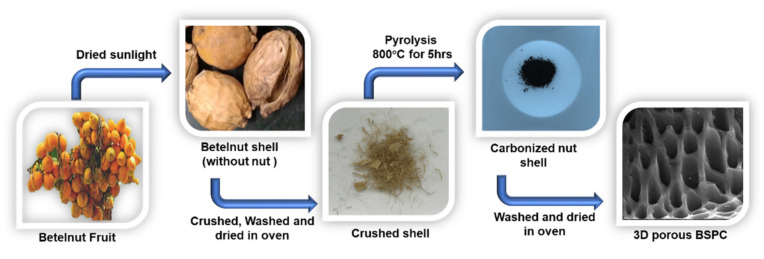
Schematic representation of the preparation of 3D hierarchical porous BSPC.

Betelnut tree (also known as betel palm, Indian nut, pinang palm, or areca catechu) samples were collected from Kumbakonam, Tamil Nadu, India. Three-dimensional hierarchical porous carbon material was prepared using betelnut waste shell as a precursor. The nut was removed, and the betelnut waste shell was dried in sunlight over 2 days. After drying, the shell was thoroughly washed with deionized water and then dried at 100 °C for 2 h in a convection oven. Subsequently, the washed betelnut shell (5.0 g) was collected in a ceramic boat. Then, the ceramic boat containing the carbon precursor was transferred into the center of a tubular furnace, which was heated to 800 °C at a heating rate of 10 °C min^−1^ under N_2_ atmosphere, followed by carbonization at 800 °C for 5 h. After the direct pyrolysis, the furnace was left to cool under N_2_ gas flow to prevent oxidation. The resulting materials were obtained after washing with DI water, acid treatment of HCl, filtering, and drying overnight at 90 °C. The resultant carbon materials were turned ‘betelnut shell-derived porous carbon’ (BSPC). A schematic for the fabrication of BSPC is provided as Figure 1.

### 2.2. Physical Characterization

A wide-angle Powder XRD pattern of the calcined carbon materials was taken using a Rigaku Miniflex II diffractometer with Cu Kα as the radiation source, at a wavelength of 0.154 nm, with a 2θ angle ranging from 10° to 80°, and a 0.02 step size. Raman spectroscopy with a 532 nm laser (Witec Alpha-300) as the excitation source in the range of 500 to 2500 cm^−1^ was used for the measurements. The BET-N_2_ adsorption and desorption isotherms were measured using a surface area and porosity analyzer (Micromeritics ASAP 2020 Accelerated Surface Area and Porosimetry system) at 77 K. Before the analysis, the samples were oven dried at 150 °C and evacuated for 12 h at 200 °C under a vacuum. The surface area was calculated using the BET method [29]. Thermogravimetric analysis (TGA) was performed using TGA-SDT Q600 in an inert atmosphere with a flow rate of 100 mL min^−1^. The morphological features were analyzed using a field-emission-scanning electron microscope (FESEM, FEI Quanta 400, FEI Company, Hillsboro, OR, USA). The carbon samples were mounted via conductive carbon tape. A thin (ca.10 nm) coating of gold sputter was deposited onto the carbon samples to decrease the charging effect. JEOL JEM-2000 high resolution transmission electron microscopy (HRTEM, JEOL Ltd., Akishima, Tokyo) was employed to obtain the micrographs.

### 2.3. Electrochemical Characterizations

The electrochemical properties of the BSPC were estimated by cyclic voltammetry (CV), galvanostatic charge–discharge (GCD), and electrochemical impedance spectroscopy (EIS) analysis using a Biologic SP-300 modular-research-grade potentiostat/galvanostat/FRA instrument. The working electrode was fabricated using BSPC material mixed with carbon black (Super-P, conductive material) and polytetrafluoroethylene (PTFE, as a binder) in a weight ratio of 75:15:10, followed by the addition of N-methyl 2-pyrollidone to form a slurry. After a thorough grinding, the slurry was coated onto a carbon sheet (area 1 cm^2^) to be used as a working electrode. This working electrode was then dried at 90 °C for 8 h in a hot oven. The electrochemical performances of the BSPC electrodes were studied in a three-electrode configuration using 1 M KOH as the electrolyte. BSPC, Hg/HgO_4_, and platinum wire served as a working electrode, reference electrode, and counter electrode (three-electrode system), respectively. The CV and GCD experiments were carried out in the potential range of −1.0 to 0.2 V at various scan rates (10 to 100 mV s^−1^) and different current densities (1, 2, 3, 4, and 5 A g^−1^). The EIS measurements were performed by applying a voltage with 5 mV amplitude in a frequency range from 10^−2^ to 10^5^ Hz to the open circuit potential. The specific capacitance values of the BSPC were calculated from the discharge curve using the following equation:

(1) $$C_{s}=\frac{I\;\times \;\Delta t}{m\;\times \;\Delta V}$$

in which *C_s_* is the specific capacitance (F g^−1^), *I* is the applied current, Δ*t* is the discharge time (s), *V* is the working potential window, and *m* is the mass of the active material [30]. Two BSPC-based symmetrical SC cells were assembled with 1 M KOH as the aqueous electrolyte and evaluated over the voltage window of 0.0 to 0.6 V. Likewise, in case of full-cell symmetrical SC, the specific capacitance, energy density, and power density were evaluated as follows:

(2) $$C_{S}=2\;\times \;\frac{I\;\times \;\Delta t}{m\;\times \;\Delta V}$$

(3) $$E_{D}=0.5\;\times \;\frac{C_{full\;cell\;}\;\times \;{\left(\Delta V\right)}^{2}}{3.6}$$

(4) $$P_{D}=\frac{E_{D}\;\times \;3600}{\Delta t}$$

## 3. Results and Discussion

### 3.1. Structural, Physical and Morphological Studies

**Figure 2:**
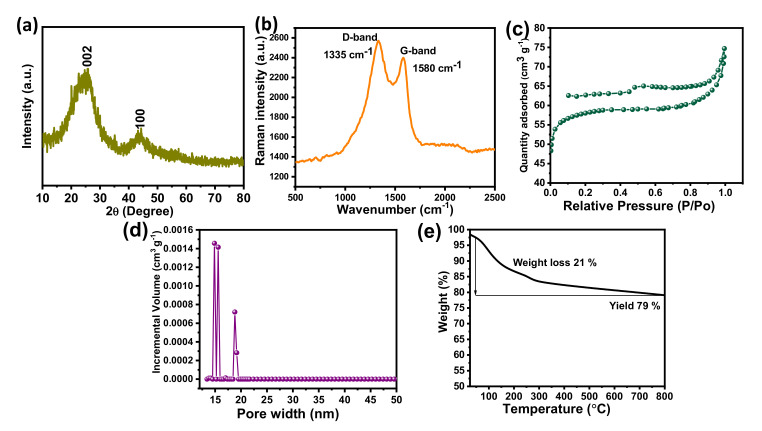
(**a**) X-ray diffraction, (**b**) Raman spectrum, (**c**) N_2_ adsorption/desorption isotherms, (**d**) pore size distribution, and (**e**) TGA analysis of the BSPC.

The X-ray diffraction pattern of the BSPC is shown in Figure 2a. The pattern reflects two broad peaks around 25.8° and 44.5° which correspond to the (002) and (100) planes, suggesting the amorphous nature of the carbon framework. No additional peaks were observed, indicating that the acid washing completely removed the other metals and elements from the samples. These results suggest the successful preparation of high-purity carbon material from betelnut-shell waste [31]. Figure 2b shows the Raman spectra of BSPC. The BSPC exhibits two broad overlapping bands around 1336 and 1582 cm^−1^, which are commonly designated as D and G bands and correspond to sp^2^-bonded carbon atoms. The structural defects and disordered nature reveal the enhancement of graphitization through carbonization.

The nitrogen adsorption/desorption isotherms of BSPC were measured to determine their specific surface areas (Figure 2c). Consistent with the classification of the International Union of Pure and Applied Chemistry (IUPAC), the N_2_ adsorption/desorption isotherms of BSPC showed a type IV isotherm with a hysteresis loop, which is a characteristic feature of porous carbon nanomaterials [32]. The isotherms showed a H3 hysteresis loop, suggesting the presence of slit-shaped pores. The pore-size distributions (PSD) were determined using a nonlocal density functional theory (NLDFT) slit/cylindrical-shaped pore-equilibrium model of the BSPC (Figure 2d). The results confirmed that the BSPC has a hierarchical porous structure with dominance of mesopores, which contributes to its fast charge/discharge capabilities and facilitated charge transport. BSPC exhibits a high specific surface area of 425 m^2^ g^−1^ and total pore volume of 0.46 cm^3^ g^−1^, in addition to a pore width of 16 nm. The high specific surface area and high volume of pores are helpful for the adsorption/desorption of charges during electrochemical processes. The high surface area of BSPC results in enhanced electrode-surface exposure to the electrolytic ions, inducing well interaction and, thus, high super capacitive performance.

A TGA analysis was performed to determine the pyrolysis temperature of BSPC (Figure 2e). The notable weight loss of 21 wt% was observed at around 100 to 336 °C, due to the loss of superficial moisture and volatile matters (CO_2_, H_2_, etc.) and the removal of physisorbed water moisture from the betelnut shell. The betelnut shell had potentially changed to carbon material for high yield and high stability with a char yield of 79 wt% at 800 °C. These results reveal that the betelnut shell is a suitable precursor to produce thermally stable renewable porous carbon materials with high yield.

**Figure 3:**
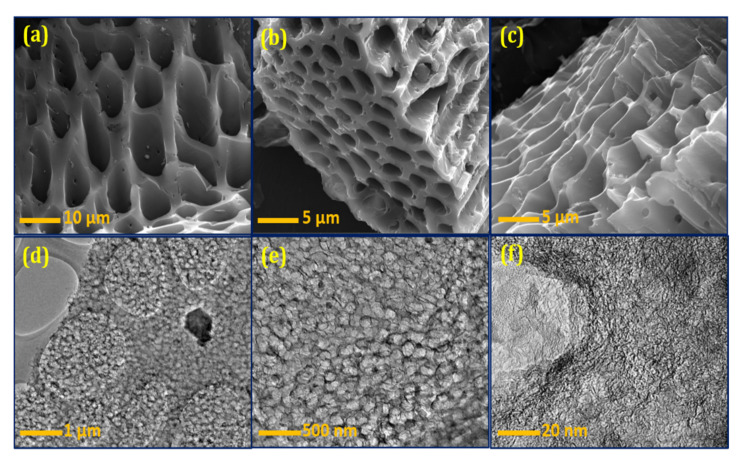
(**a**–**c**) FESEM and (**d**–**f**) HRTEM images of the BSPC at different magnifications.

Figure 3a–c shows the FESEM images of the BSPC. A honeycomb-shaped porous structure with well-organized pores is clearly observed (Figure 3a). The different magnifications show all dimensions of material morphology with clear connected porous arrays (Figure 3b,c). The 3D hierarchical porous morphology of BSPC is likely to be highly favorable, as it offers a large interfacial area for energy-storage reaction kinetics. The HRTEM results further support this analysis of the internal morphological features of BSPC (Figure 3d–f). The BSPC showed a honeycomb-like interconnected porous assembly (Figure 3d,e) at different levels of magnification. Interestingly, the high magnification of HRTEM images in Figure 3f revealed numerous interconnected micropores of BSPC. As observed by the Raman spectrum, the higher carbonization temperature of the biomass precursor created 3D hierarchical porous structures, with disordered carbons comprising a comparatively large number of defects. The honeycomb-shaped hierarchical porosity of the BSPC provides continuous paths for charges, potentially enhancing its electrochemical energy-storage capacity [33,34].

### 3.2. Electrochemical Studies and Results

**Figure 4:**
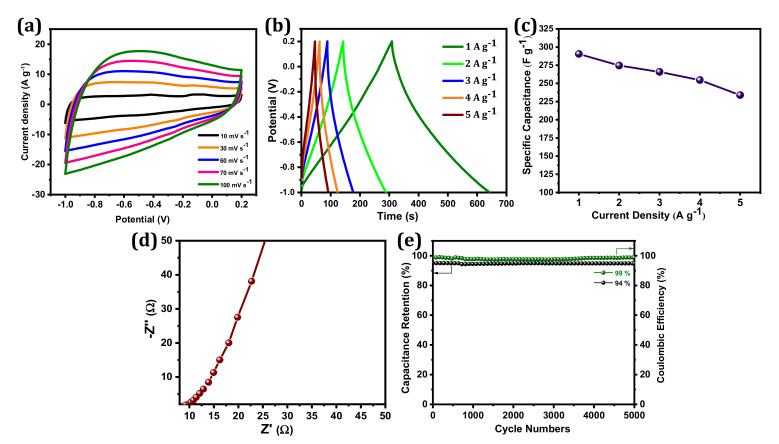
Electrochemical characterization of BSPC electrode: (**a**) CV curves at the scan rates of 10 to 100 mV s^−1^, (**b**) GCD curves at the specific current densities of 1 to 5 A g^−1^, (**c**) the specific capacitances at different specific currents ranging from 1 to 5 A g^−1^, (**d**) Nyquist plots with an amplitude of 10 mV s^−1^ over a frequency range of 10^−2^ to 10^5^ Hz, (**e**) cyclic stability and coulombic efficiency at a specific current of 5 A g^−1^ over 5000 GCD cycles.

The electrochemical performances of BSPC were investigated by CV and GCD measurements in a 1 M KOH electrolyte with a three-electrode configuration. In Figure 4a, the BSPC electrode displayed a quasi-rectangular shape in CV curves, which is typical of an electric double-layer capacitor (EDLC). This behavior indicates good ion transport across the interconnected porous networks of BSPC, owing to the increased accessibility of the electrolyte ions to the inner active area of electrodes. The 3D porous structure of BSPC can play a crucial role in increasing capacitive performance at high charge–discharge rates and enabling the circulation of electrolyte ions across the electrode surface [35]. Thus, the BSPC retains a similar CV shape even at scan rates from 10 mV s^−1^ to 100 mV s^−1^, demonstrating its high rate-retention capability. This was further corroborated by GCD studies (Figure 4b). The GCD profiles of the BSPC electrode reflect significant electrochemical properties with a nearly symmetric triangular shape without a notable IR drop even at current densities as high as 5 A g^−1^. However, the discharging times of GCD curves decreased as current densities increased [36]. The BSPC electrode delivered a high specific capacitance of 290 F g^−1^ at a specific current of 1 A g^−1^, which was calculated using the discharge curves and Equation (1) (Figure 4c).

The EIS was used to confirm the frequency response characteristics of the BSPC electrodes (Figure 4d). A nearly vertical line at the low-frequency region was obtained, which represents the dominance of EDLC [37]. Charge/discharge cyclic stability is an additional significant parameter that must be considered prior to the manufacture of SCs for commercial applications. BSPC possesses exceptional electrochemical stability; 94% cyclic retention of initial capacitance at a current density of 5 A g^−1^ over at a minimum of 5000 GCD cycles, and the coulombic efficiency of BSPC is approximately 99% (Figure 4e). Interestingly, the mesoporous nature of the carbon material helped to expand the ion and charge storage capacities. Likewise, the micropores decreased the ion diffusion length while the macropores acted as ion transfer channels. The 3D hierarchical pores provided the high specific surface area of the carbon electrode that enhanced the electrical double layer, improving specific capacitance. These aforementioned properties suggest that BSPC shows potential for SC applications.

**Figure 5:**
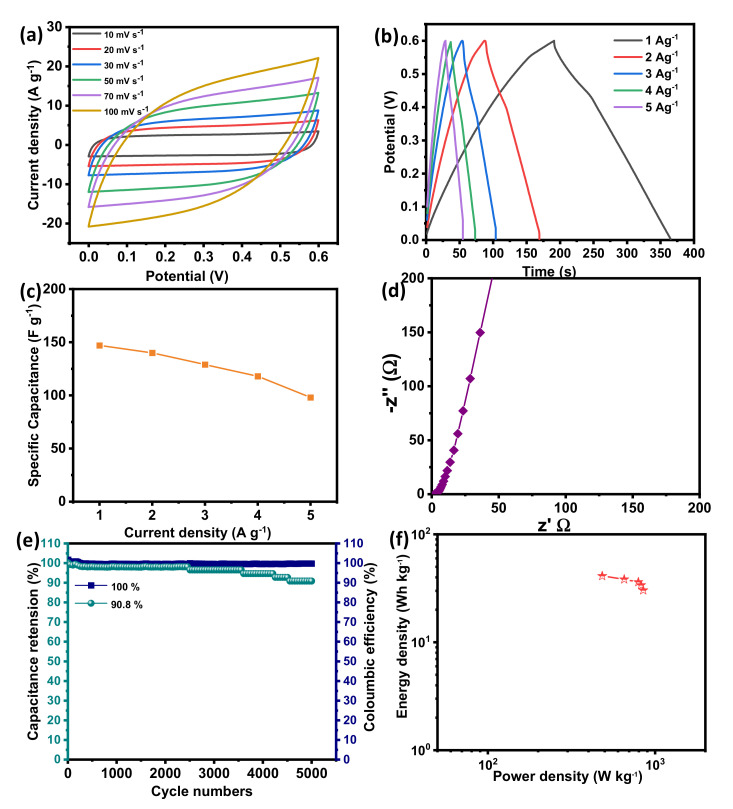
Electrochemical characterization of BSPC electrode in a two-electrode system. (**a**) CV curves at the scan rates of 10 to 100 mV s^−1^. (**b**) GCD curves at the specific current densities of 1 to 5 A g^−1^. (**c**) The specific capacitances at different specific currents ranging from 1 to 5 A g^−1^. (**d**) Nyquist plots with an amplitude of 10 mV s^−1^ over a frequency range of 10^−2^ to 10^5^ Hz. (**e**) Cyclic stability and coulombic efficiency at a specific current of 5 A g^−1^ over 5000 GCD cycles. (**f**) Ragone plot.

To study the electrochemical performances in terms of practical applications, the symmetrical SC in a two-electrode system was assembled using two BSPC electrodes in 1 M KOH as an aqueous electrolyte. Figure 5a shows the CV curves of BPSCs with a different scan rate of 10 to 100 mVs^−1^ over the voltage window of 0 to 0.6 V. The shape of CV curves is quasi-rectangular with no other peaks, confirming the EDLC performance of a symmetric full-cell SC. Figure 5b shows the GCD measurement of BPSCs at the various specific currents of 1 to 5 A g^−1^. The GCD profiles are further confirmed by the symmetrical triangular shape, which signifies the EDLC performance which supports CV measurements. Two BSPC electrodes show specific capacitance as large as 148 F g^−1^ at the current density of 1 A g^−1^ (Figure 5c). The lower frequency region of EIS showing a vertical line is further confirmed by the governance of EDLC performance in a two-electrode symmetrical system (Figure 5d). Furthermore, they exhibit a good cyclic stability of 90.8 % and a coulombic efficiency of almost 100% obtained at the current density of 5 A g^−1^ over 5000 charge/discharge cycles, demonstrating the formation of a stable electrode/electrolyte interface.

Figure 5f shows the Ragone plot. Symmetric BSPCs show the maximum energy density of 41 Wh Kg^−1^ and power density of 483 W Kg^−1^. This value is comparable to or even exceeds the values of other literature regarding biomass-derived SC electrodes (Table 1).

## 4. Conclusions

We demonstrated that the biomass waste from a betelnut shell can be transformed into a potential electrode for high-performance SC. The 3D hierarchical porous carbon material was effectively produced by a direct carbonization process applied to the biomass waste without any chemical activation process. The electrode with a unique 3D hierarchical porous network structure, which has a high specific surface area, showed a specific capacitance of 290 F g^−1^ at a specific current of 1 A g^−1^ in 1 M KOH. Furthermore, BSPC offered an excellent charge/discharge cyclic stability of 94% at a current density of 5 A g^−1^ over at least 5000 GCD cycles. However, the symmetric system demonstrates the specific capacitance of 148 F g^−1^ at 1 A g^−1^, and it exhibits high cyclic stability of 90.8% over 5000 GCD cycles with a high energy density of 41 Wh Kg^−1^ and a power density of 483 W Kg^−1^ in an aqueous 1 M KOH electrolyte. This work provides a new insight to produce biomass-derived porous carbon material-based electrodes for the application of high-performance SCs with high cyclic stability and durability.

## Figures and Tables

**Table 1:** The comparison of various biomass-derived carbon materials and their supercapacitive performance in the literature.

Electrode	Specific Surface Area (m^2^ g^−1^)	Electrolyte	Specific Capacitance (F g^−1^)	Energy Density (Wh Kg^−1^)	Power Density (W Kg^−1^)	References
Betelnut shell	425	1M KOH	290	41	483	This work
Cucumber	389	6 M KOH	143	6.5	45.2	[38]
Pig nails	2569	6 M KOH	231	7	500	[39]
Banana peel	1357	6 M KOH	210.6			[40]
Lignin	1440	6 M KOH	268	40.89		[41]
Coconut shell	1998	1.5M H_2_SO_4_	132.3	92.1	2046.9	[42]
Chinar fruit	1758	6 M KOH	247.5	21.99	400	[43]
Peony pollen	824	6 M KOH	209			[44]
Chitin	2631	1 M H_2_SO_4_	245	7.9	0.12	[45]
Corn grains	3199	6 M KOH	257			[46]
Firewood	1130	1 M HNO_3_	142			[47]

## Data Availability

Not applicable.
